# Distinct phenotypes of multisystem inflammatory syndrome in children: a cohort study

**DOI:** 10.1186/s12969-023-00815-w

**Published:** 2023-04-12

**Authors:** Thomas Renson, Nils D. Forkert, Kimberly Amador, Paivi Miettunen, Simon J. Parsons, Muhammed Dhalla, Nicole A. Johnson, Nadia Luca, Heinrike Schmeling, Rebeka Stevenson, Marinka Twilt, Lorraine Hamiwka, Susanne Benseler

**Affiliations:** 1 Rheumatology, Department of Pediatrics, Alberta Children’s Hospital, University of Calgary Cumming School of Medicine, 28 Oki Drive NW, Calgary, AB T3B 6A8 Canada; 2 Nephrology, Department of Pediatrics, Alberta Children’s Hospital, University of Calgary Cumming School of Medicine, Calgary, AB Canada; 3 Department of Radiology, University of Calgary Cumming School of Medicine, Calgary, AB Canada; 4 Alberta Children’s Hospital Research Institute, University of Calgary Cumming School of Medicine, Calgary, AB Canada; 5 Critical Care Medicine, Department of Pediatrics, Alberta Children’s Hospital, University of Calgary Cumming School of Medicine, Calgary, AB Canada

**Keywords:** MIS-C, COVID-19, Phenotypes, Cardiogenic shock, Pediatric intensive care

## Abstract

**Background:**

Multisystem inflammatory syndrome in children (MIS-C) is a severe disease with an unpredictable course and a substantial risk of cardiogenic shock. Our objectives were to (a) compare MIS-C phenotypes across the COVID-19 pandemic, (b) identify features associated with intensive care need and treatment with biologic agents.

**Methods:**

Youth aged 0–18 years, fulfilling the World Health Organization case definition of MIS-C, and admitted to the Alberta Children’s Hospital during the first four waves of the COVID-19 pandemic (May 2020-December 2021) were included in this cohort study. Demographic, clinical, biochemical, imaging, and treatment data were captured.

**Results:**

Fifty-seven MIS-C patients (median age 6 years, range 0–17) were included. Thirty patients (53%) required intensive care. Patients in the third or fourth wave (indicated as phase 2 of the pandemic) presented with higher peak ferritin (µg/l, median (IQR) = 1134 (409–1806) vs. 370 (249–629), *P* = 0.001), NT-proBNP (ng/l, median (IQR) = 12,217 (3013–27,161) vs. 3213 (1216–8483), *P* = 0.02) and D-dimer (mg/l, median (IQR) = 4.81 (2.24–5.37) vs. 2.01 (1.27–3.34), *P* = 0.004) levels, and higher prevalence of liver enzyme abnormalities (n(%) = 17 (68) vs. 11 (34), *P* = 0.02), hypoalbuminemia (n(%) = 24 (100) vs. 25 (81), *P* = 0.03) and thrombocytopenia (n(%) 18 (72) vs. 11 (34), *P* = 0.007) compared to patients in the first two waves (phase 1). These patients had a higher need of non-invasive/mechanical ventilation (n(%) 4 (16) vs. 0 (0), *P* = 0.03). Unsupervised clustering analyses classified 47% of the patients in the correct wave and 74% in the correct phase of the pandemic. NT-proBNP was the only significant contributor to the need for intensive care in all applied multivariate regression models. Treatment with biologic agents was significantly associated with peak CRP (mg/l (median, IQR = 240.9 (132.9-319.4) vs. 155.8 (101.0-200.7), *P* = 0.02) and ferritin levels (µg/l, median (IQR) = 1380 (509–1753) vs. 473 (280–296)).

**Conclusions:**

MIS-C patients in a later stage of the pandemic displayed a more severe phenotype, reflecting the impact of distinct SARS-CoV-2 variants. NT-proBNP emerged as the most crucial feature associated with intensive care need, underscoring the importance of monitoring.

**Supplementary Information:**

The online version contains supplementary material available at 10.1186/s12969-023-00815-w.

## Introduction

In December 2019, the world was caught off-guard by the appearance of the early reports concerning the severe acute respiratory syndrome coronavirus 2 (SARS-CoV-2)-associated coronavirus disease 2019 (COVID-19) in Wuhan, China [1]. Notwithstanding their striking underrepresentation in the epidemiology of acute COVID-19 [2], a proportion of children have developed a severe multisystem inflammatory syndrome (MIS-C) following infection. This disease entity was first observed in April 2020 in London, United Kingdom, one of the European epicenters of the initial COVID-19 outbreak [3]. Patients from this small MIS-C cohort were characterized by the constellation of fever, circulatory shock, and laboratory evidence of hyperinflammation. Subsequent MIS-C case series from Bergamo (Italy) and Paris (France) confirmed the frequent occurrence of Kawasaki disease (KD)-like mucocutaneous manifestations, including rash, conjunctivitis, erythematous and/or crackled lips, strawberry tongue, and extremity changes [4, 5]. Notwithstanding the relatively low mortality rate (1.9%) [6], MIS-C patients can present with severe left ventricular dysfunction leading to life threatening circulatory shock and multi-organ failure [3, 7−9]. Importantly, the evolution to cardiogenic shock may occur quickly and unpredictably. Up to three quarters of MIS-C cases require admission to a pediatric intensive care unit (PICU) for respiratory and/or circulatory support [6]. Several risk factors for PICU admission have been identified, including age (≥ 6 years), ethnicity (Black patients), respiratory symptoms, gastrointestinal involvement, and certain laboratory features, including high C-reactive protein (CRP), troponin, ferritin, D-dimer, N-terminal B-type natriuretic peptide (NT-proBNP), interleukin (IL)-6 levels, thrombocytopenia, and lymphopenia [10]. The optimal management of MIS-C patients, including predictors of PICU admission and the indications for the use of biologic agents, is still equivocal. Furthermore, the evolution of the clinical phenotype of MIS-C triggered by different COVID-19 variants remains unexplored. The aims of the current study were therefore to (a) describe a single center cohort of children with MIS-C warranting admission to a tertiary hospital, (b) compare clinical, laboratory, and imaging features, as well as treatment between MIS-C patients presenting during the different COVID-19 waves, (c) identify factors associated with severe disease requiring PICU admission, and (d) identify factors associated with treatment with biologic agents, in addition to intravenous immunoglobulins (IVIG).

## Methods

### Study design and subjects

A single center cohort study of children with MIS-C, diagnosed between May 2020 and December 2021 was performed at the Alberta Children’s Hospital. Patients were included with a diagnosis of MIS-C according to the World Health Organization case definition [11]. Patients were excluded if the diagnosis was revised during or after hospital admission. Patients were eligible to be included in the final analyses if both case reviewers (TR and SB) agreed on the diagnosis of MIS-C.

### Demographic and clinical characteristics

Captured demographic characteristics included date of birth and sex. Clinical features investigated consisted of past medical history, clinical signs and symptoms at MIS-C presentation, laboratory findings, echocardiogram features (ventricular dysfunction, coronary dilation/ectasia/aneurysm), chest X-ray findings, and treatment. MIS-C clinical signs and symptoms included fever, extremity changes (redness, swelling, desquamation), rash, bilateral conjunctivitis without exudate, lip and/or oral cavity changes (erythematous/crackled lips, strawberry tongue with erythema and prominent fungiform papillae, erythema of the oropharyngeal mucosa), cervical lymphadenopathy (> 1.5 cm), gastrointestinal manifestations (abdominal pain, diarrhea, vomiting), and clinical signs of hypotension and/or circulatory shock. Fulfillment of the American Heart Association (AHA) diagnostic criteria for KD was assessed [12]. Collected laboratory findings included peak levels of CRP, ferritin, partial thromboplastin time, D-dimers, troponin, NT-proBNP, liver enzyme abnormalities, and nadir levels of sodium, albumin, and platelet counts. MIS-C patients admitted from May 2020 until the end of August 2020 were categorized in the first wave of the COVID-19 pandemic, from September 2020 to the end of March 2021 in the second wave, from April 2021 to the end of July 2021 in the third wave, and from August 2021 until the end of December 2021 in the fourth wave.

### Outcome

The primary outcome was PICU admission. In-hospital criteria for PICU admission were refractory hypotension requiring circulatory support with vasopressors, respiratory failure requiring respiratory support with non-invasive ventilation or invasive mechanical ventilation, and/or severe refractory shock requiring extracorporeal membrane oxygenation. Secondary outcome was treatment with biologic agents, in addition to IVIG.

### Statistical analyses

Descriptive statistics were applied to describe demographic and clinical features. Mann-Whitney U test, Kruskal-Wallis test, and Fisher’s exact tests were used to assess the differences in MIS-C features between patient subgroups. Multiple logistic regression models encompassing three covariates (considering the limited sample size) explored factors significantly associated with the need for intensive care. These covariates were selected based on univariate analyses (Mann-Whitney U test and Fisher’s exact tests). Correlations between peak NT-proBNP levels and other MIS-C features were assessed by Mann-Whitney U tests and Spearman’s correlation coefficients. Hypothesis tests were two-sided. *P* values < 0.05 were considered as statistically significant and are displayed in the tables in bold. REDCap was used as a data capture tool. Data analysis was performed in IBM SPSS Statistics v25. Figures were created in R Studio.

In addition to conventional statistical analyses, unsupervised machine learning was used to identify distinct groups in the data and investigate how well these groups correspond to the waves. Briefly described, unsupervised machine learning aims to uncover patterns without making use or requiring corresponding ground truth data. These methods can automatically identify clusters of similar cases (groups) within a dataset [13, 14]. In this work, self-organizing maps were used to identify clusters in the data. A self-organizing map is a type of artificial neural network that can compute a low-dimensional representation of a higher dimensional dataset, while conserving the underlying structure of its input space [15]. The self-organizing maps used in this work were optimized using standard hyperparameters including a learning rate of 1.0, 1000 epochs for convergence, and 2000 epochs for ordering.

## Results

### Demographic and clinical characteristics of MIS-C patients

Sixty-two patients were included. Five patients were subsequently excluded based on a revised diagnosis. These diagnoses included KD, systemic-onset juvenile idiopathic arthritis, viral-induced myositis and myocarditis, inflammatory myositis of unknown origin, and bacterial sepsis with multiple epidural abscesses. Fifty-seven MIS-C patients were included in the final analysis (Table 1). The median age was 6 years (range 0–17). The majority of the patients were male (0.72) and this predominance was maintained across the different waves of the COVID-19 pandemic. Forty-seven patients (0.82) had laboratory evidence of previous SARS-CoV-2 exposure. None of the patients died during their hospitalization. Thirty patients (0.53) needed intensive care for advanced cardiovascular and/or respiratory management. Patients warranting PICU admission were older compared to those managed on the general ward (median (IQR) 7 (5–9) years versus 5 (2–8) years respectively, Mann-Whitney U value (*U)* = 277.5, *P* = 0.04), whereas there was no difference regarding sex (24 male patients (0.59) versus 6 female patients (0.38) respectively; *P* = 0.24). Less than 10% of the patients had a chronic comorbidity: three patients (0.05) had well-controlled asthma without maintenance treatment, two patients (0.04) had a history of attention-deficit/hyperactivity disorder. Twenty-two MIS-C patients (0.39) fulfilled the AHA diagnostic criteria for typical KD. All patients had at least one out of five principal clinical features of KD, and a vast majority (0.91) had three or more features. The most prominent KD feature in our population was bilateral conjunctivitis, occurring in 53 patients (0.93), whereas the least frequent feature was cervical lymphadenopathy (0.16). Nearly all MIS-C patients (0.97) had gastrointestinal manifestations. Fifty patients (0.88) presented with signs of circulatory shock and/or hypotension. Cardiac involvement was frequently observed with high median peak values of serum NT-proBNP (7010 ng/l) and troponin (32.0 ng/l), and approximately one third of the patients having left ventricular dysfunction on echocardiogram. Hyperinflammation was a cardinal feature of our MIS-C population with high median peak CRP (188.3 mg/l) and ferritin (482 µg/l) levels. Moreover, hypoalbuminemia (0.86), hyponatremia (0.63), and thrombocytopenia (0.51) were key laboratory findings. All patients received initial treatment with IVIG. Forty-four patients (0.77) were treated with corticosteroids, of which 40 patients (0.91) initially received intravenous pulse steroids. Eight patients (0.14) received a biologic agent, in addition to IVIG: five patients were treated with IL-1 inhibition (anakinra), two with IL-6 inhibition (tocilizumab) (of which one patient also received anakinra), and two with TNFα-inhibition (infliximab). Both patients treated with infliximab presented in the first wave, when it was unclear how to manage MIS-C. Those patients were therefore treated per KD guidelines.

**Table 1:** Demographic and clinical features of MIS-C patients across the COVID-19 waves

Demographic characteristics					
	**All waves** (n = 57)	**Wave 1** (n = 7)	**Wave 2** (n = 25)	**Wave 3** (n = 17)	**Wave 4** (n = 8)
Male sex (n, %)	41 (72)	5 (71)	18 (72)	14 (82)	5 (63)
Age at admission, years (median, IQR)	6 (4–8)	7 (1–8)	5 (3–8)	7 (4–9)	7 (5–10)
**Clinical characteristics**					
	**All waves** (n = 57)	**Wave 1** (n = 7)	**Wave 2** (n = 25)	**Wave 3** (n = 17)	**Wave 4** (n = 8)
***History***					
Prematurity (n, %)	1 (2)	0	0	0	1 (13)
Chronic comorbidity (n, %)	5 (9)	2 (29)	1 (4)	2 (12)	0
***Clinical signs and symptoms***					
Fulfillment of AHA Kawasaki disease’s criteria (n, %)	22 (39)	3 (43)	13 (52)	5 (29)	1 (13)
Days of fever at admission (median, IQR)	5 (3–6)	5 (4–15)	5 (3–6)	4 (4–5)	4 (3–5)
Total days of fever (median, IQR)	5 (4–7)	5 (5–15)	6 (5–7)	5 (4–6)	5 (4–7)
Extremity changes (n, %)	42 (74)	5 (71)	20 (80)	13 (76)	4 (50)
Skin rash (n, %)	48 (84)	7 (100)	24 (96)	13 (76)	4 (50)
Bilateral conjunctivitis without exudate (n, %)	53 (93)	6 (86)	25 (100)	15 (88)	7 (88)
Changes lips and/or oral cavity (n, %)	41 (72)	5 (71)	21 (84)	12 (71)	3 (38)
Cervical lymphadenopathy (> 1.5 cm) (n, %)	9 (16)	1 (14)	5 (20)	1 (6)	2 (25)
Gastrointestinal manifestations (n, %)	55 (97)	7 (100)	25 (100)	15 (88)	8 (100)
Shock/hypotension (n, %)	50 (88)	4 (57)	24 (96)	15 (88)	7 (88)
***Laboratory features during hospitalization***					
Microbiological or serological evidence for SARS-CoV-2 exposure (n, %)^1^	47 (82)	1 (14)	22 (88)	17 (100)	7 (88)
Coagulation dysfunction (INR > 1.1) (n, %)	47 (82)	7 (100)	18 (72)	14 (82)	8 (100)
INR value (median, IQR)	1.4 (1.3–1.5)	1.4 (1.2–1.5)	1.4 (1.3–1.5)	1.4 (1.3–1.7)	1.3 (1.2–1.8)
Peak C-reactive protein, mg/l (median, IQR)	188.3 (105.0-234.3)	200.0 (96.0-234.0)	142.0 (89.0-187.5)	201.0 (127.7-244.7)	151.8 (72.0-302.3)
Peak ferritin, µg/l (median, IQR)	482 (315–1164)	376 (247–512)	417 (242–746)	516 (451–1853)	1426 (627–2048)
Peak D-dimer, mg/l (median, IQR)	3.42 (2.04–4.81)	2.20 (1.08–3.27)	2.26 (1.28–4.22)	4.15 (2.23–5.36)	5.41 (2.73–6.07)
Peak troponin, ng/l (median, IQR)	32.0 (15.0–72.0)	7.0 (6.0-13.8)	38.0 (19.0–69.0)	40.0 (16.0–73.0)	52.0 (20.8–79.5)
Peak NT-proBNP, ng/l (median, IQR)	7010 (1961–14,852)	1217 (973–4810)	4022 (1684–9790)	12,983 (4901–30,302)	12,901 (927-25493)
Liver enzyme abnormalities (n, %)	28 (49)	3 (43)	8 (32)	11 (65)	6 (75)
Hyponatremia < 133 mmol/l (n, %)	36 (63)	2 (29)	17 (68)	11 (65)	6 (75)
Sodium nadir, mmol/l (median, IQR)	132 (129–134)	133 (130–136)	132 (129–134)	132 (128–134)	131 (129–134)
Hypoalbuminemia < 30 g/l (n, %)	49 (86)	6 (86)	19 (76)	16 (94)	8 (100)
Albumin nadir, g/l (median, IQR)	20 (18–25)	21 (18–24)	24 (20–29)	19 (16–20)	23 (18–25)
Thrombocytopenia < 150 × 10E9/l (n, %)	29 (50.9)	1 (14)	10 (40)	11 (65)	7 (88)
Platelet count nadir, x10E9/l (median, IQR)	147 (115–230)	262 (172–364)	176 (124–315)	140 (120–174)	106 (58–148)
***Additional investigations***					
Concurrent other viral infection (n, %)	4 (7)	0	2 (8)	1 (6)	1 (13)
Concurrent bacterial infection (with microbiological evidence) (n, %)	2 (4)	0	1 (4)	1 (6)	0
Coronary ectasia, dilatation and/or aneurysm on cardiac US (n, %)	8 (14)	3 (43)	3 (12)	2 (12)	0
Ventricular dysfunction on cardiac US (n, %)	20 (35)	0	9 (36)	7 (41)	4 (50)
Chest X-ray abnormalities (n, %)	21 (37)	3 (43)	8 (32)	6 (35)	4 (50)
***Management***					
IVIG (n, %)	57 (100)	7 (100)	25 (100)	17 (100)	8 (100)
Corticosteroids (n, %)	44 (77)	3 (43)	18 (72)	15 (88)	8 (100)
Intravenous methylprednisolone pulses (in those treated with corticosteroids) (n, %)	40 (91)	3 (100)	16 (89)	15 (100)	6 (75)
Acetylsalicylic acid (n, %)	57 (100)	7 (100)	25 (100)	17 (100)	8 (100)
Hydroxychloroquine (n, %)	0	0	0	0	0
Biologic agents (other than IVIG) (n, %)	8 (14)	2 (29)	0	4 (24)	2 (25)
Antibiotics (n, %)	52 (91)	7 (100)	24 (96)	16 (94)	5 (63)
Antiviral treatment (n, %)	1 (2)	0	0	1 (6)	0
Intensive care unit admission (n, %)	30 (53)	2 (29)	15 (60)	8 (47)	5 (63)
Vasopressors (n, %)	30 (53)	2 (29)	15 (60)	8 (47)	5 (63)
Non-invasive ventilation (CPAP, BiPAP) (n, %)	2 (4)	0	0	1 (6)	1 (13)
Mechanical ventilation (n, %)	3 (5)	0	0	3 (18)	0

^1^Positive polymerase chain reaction or antigen test in the eight weeks prior to MIS-C symptom onset and/or positive IgG before therapeutic administration of intravenous immunoglobulins. (n = number, IQR = interquartile range, AHA = American Heart Association, INR = international normalized ratio, NT-proBNP = N-terminal B-type natriuretic peptide, US = ultrasound, IVIG = intravenous immunoglobulins, CPAP = continuous positive airway pressure, BiPAP = bilevel positive airway pressure)

### Comparative analysis of MIS-C phenotypes during the COVID-19 pandemic

**Fig. 1:**
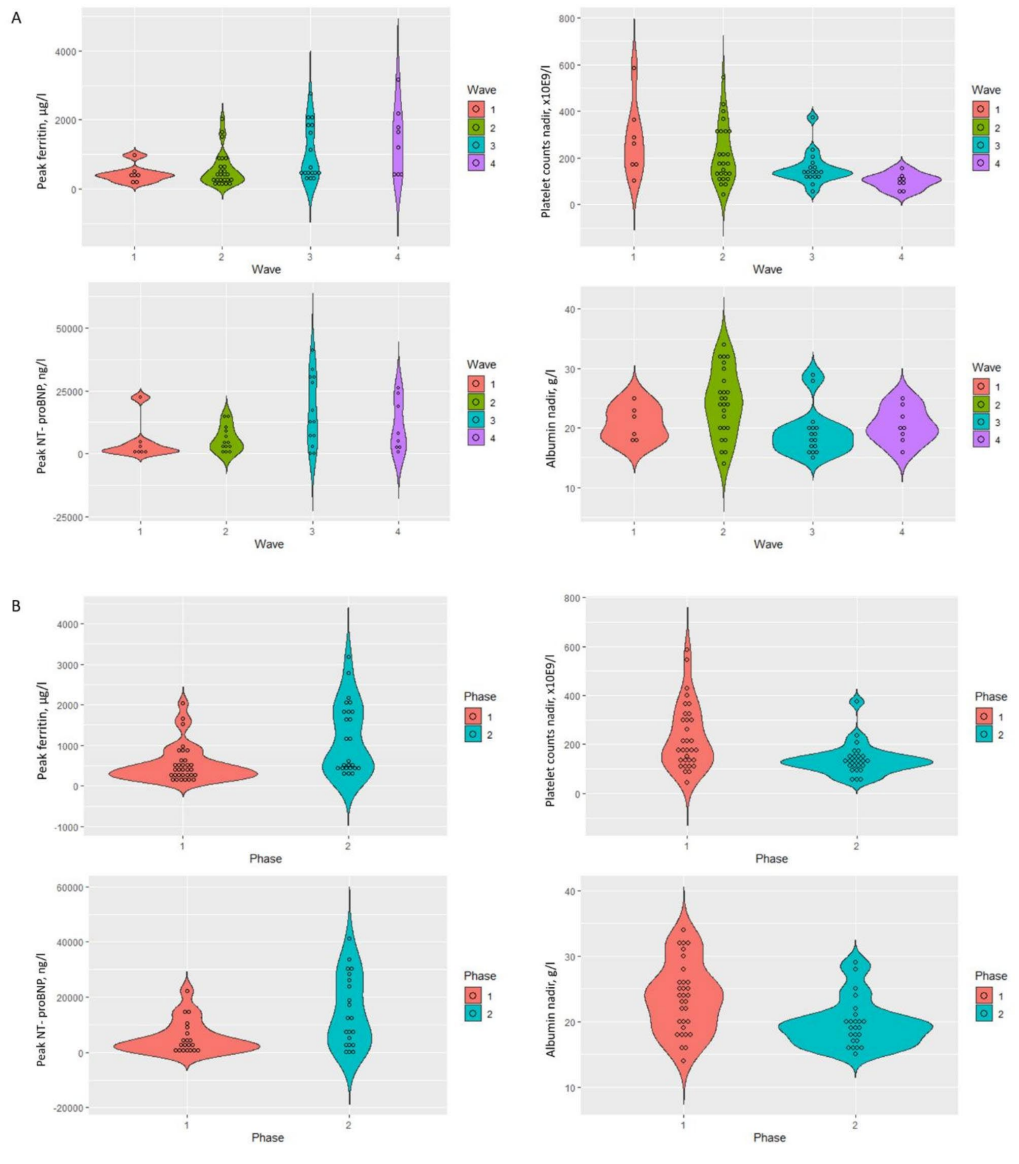
**MIS-C patients presented with distinct laboratory features across the COVID-19 pandemic.** Violin plots depicting differences in key laboratory MIS-C features in patients presenting during the four waves (panel A) and both phases (phase one is wave one and two combined, phase two is wave three and four combined; panel B) of the COVID-19 pandemic: peak ferritin values, platelet counts nadir, albumin nadir values, and peak NT-proBNP values

Most differences in MIS-C clinical and laboratory features were found when comparing patients presenting during the second versus the fourth wave, and the first versus the fourth wave (Table 2). The smallest differences were observed between patients presenting during the third and the fourth wave. Only one MIS-C patient in the fourth wave fulfilled the criteria for typical KD. Moreover, three out of four patients with only one principal KD feature presented during the fourth wave. Fisher’s exact and Kruskal-Wallis tests did not observe any significant differences regarding the other MIS-features in Table 1. High ferritin and NT-proBNP values and low albumin values and platelet counts were mainly observed in MIS-C patients presenting during the third and fourth wave (Fig. 1).

The above results led us to hypothesize that MIS-C patients presenting during the third and fourth wave had a distinct clinical and biochemical phenotype compared to those encountered during the prior waves. Splitting the COVID-19 pandemic into two phases (wave one and two combined versus wave three and four combined) resulted in significant differences in phenotype (Table 2; Fig. 1). Patients presenting during the second phase presented with higher ferritin, D-dimer, and NT-proBNP values, more liver enzyme abnormalities, more frequent and more severe hypoalbuminemia, and more frequent and more severe thrombocytopenia. Patients from the second phase fulfilled less frequently the KD criteria compared to the first phase (0.24 versus 0.50), but the difference lacked statistical significance (*P* = 0.06).

**Table 2:** **Upper panel**: MIS-C features differed between the respective COVID-19 waves. **Lower panel**: Distinct MIS-C features in patients presenting during the first phase of the COVID-19 pandemic (first and second wave combined) compared to those presenting during the second phase (third and fourth wave combined)

MIS-C features	Wave 1 vs. 2	Wave 1 vs. 3	Wave 1 vs. 4	Wave 2 vs. 3	Wave 2 vs. 4	Wave 3 vs. 4
Shock/hypotension	**0.03**	0.13	0.28	0.56	0.43	> 0.99
Skin rash	> 0.99	0.28	0.08	0.14	**0.008**	0.36
Peak ferritin	0.83	**0.04**	**0.01**	**0.02**	**0.02**	0.55
Liver enzyme abnormalities	0.67	0.39	0.32	0.06	**0.05**	> 0.99
Peak D-dimer	0.63	0.08	0.07	**0.05**	**0.02**	0.55
Peak troponin	**0.002**	**0.02**	**0.002**	0.66	0.2	0.14
Thrombocytopenia	0.37	0.07	**0.01**	0.21	**0.04**	0.36
Platelet count nadir	0.41	**0.03**	**0.003**	0.15	**0.003**	**0.01**
Albumin nadir	0.14	0.22	0.99	**0.004**	0.08	0.24
Treatment with corticosteroids	0.2	**0.04**	**0.03**	0.27	0.15	> 0.99
Treatment with biologicals	**0.04**	> 0.99	> 0.99	**0.02**	0.05	> 0.99
	**Phase 1** (n = 32)	**Phase 2** (n = 25)	**P**			
Peak ferritin, mg/l (median, IQR)	370 (249–629)	1134 (409–1806)	**0.001** (U = 195.5)			
Liver enzyme abnormalities (n, %)	11 (34)	17 (68)	**0.02**			
Peak D-dimer, mg/l (median, IQR)	2.01 (1.27–3.34)	4.81 (2.24–5.37)	**0.004** (U = 155.0)			
Peak NT-proBNP, ng/l (median, IQR)	3213 (1216–8483)	12,217 (3013–27,161)	**0.02** (U = 121.0)			
Hypoalbuminemia (n, %)	25 (81)*	24 (100)*	**0.03**			
Albumin nadir, g/l (median, IQR)	24 (20–25)	19 (18–22)	**0.005** (U = 208.5)			
Thrombocytopenia (n, %)	11 (34)	18 (72)	**0.007**			
Platelet counts nadir, x10E9/l (median, IQR)	215 (133–316)	127 (104–155)	**0.003** (U = 218.5)			
Treatment with corticosteroids (n, %)	21 (66)	23 (92)	**0.03**			
Non-invasive/mechanical ventilation (n, %)	0	4 (16)	**0.03**			

Upper panel: Numbers in the table represent *P* values. We refer to Table 1 for absolute numbers, percentages, median values and interquartile ranges of the MIS-C features across the different waves. Lower panel: Mann-Whitney *U* values are indicated between brackets. No differences were detected regarding the other MIS-C features (data not shown). (*one missing value; NT-proBNP = N-terminal B-type natriuretic peptide, n = number, IQR = interquartile range)

The unsupervised clustering model (Kohonen’s Self-Organizing Map [15]) using age, clinical, laboratory, and imaging features as input features, without information on the respective COVID-19 wave, classified almost half of the patients (0.47) in the correct wave (eTable 1). Cluster 1 mainly consisted of patients from the third wave (5/8). This cluster was characterized by the presence of hypotension/shock, high CRP, ferritin, D-dimer and NT-proBNP levels, liver enzyme abnormalities, hypoalbuminemia, thrombocytopenia, and ventricular dysfunction. Cluster 2 comprised patients from the second (3/7), third (2/7), and fourth wave (2/7). Striking features in this cluster were younger age, coagulation dysfunction, shock/hypotension, hyponatremia, and hypoalbuminemia. Cluster 3 mainly consisted of patients from the second wave (14/22) and was characterized by the highest prevalence of KD features. Finally, cluster 4 mainly comprised patients from the second (8/20) and first (6/20) wave. This cluster was characterized by a less severe MIS-C phenotype with a lower prevalence of hypotension/shock, lower CRP, ferritin, NT-proBNP, and D-dimer levels, less hyponatremia and thrombocytopenia, no ventricular dysfunction, and few chest X-ray abnormalities compared to the other clusters. The same self-organizing map was used to categorize patients in two clusters. This model classified three quarters of the patients (0.74) in the correct phase of the pandemic (wave 1 + 2 versus wave 3 + 4) (eTable 1). Cluster 1 mainly consisted of patients from the second phase (19/28). Compared to cluster 1, striking features of cluster 2 were shock/hypotension, very high ferritin, D-dimer, and NT-proBNP levels, (severe) hypoalbuminemia, thrombocytopenia, liver enzyme abnormalities, ventricular dysfunction, and chest X-ray abnormalities.

### MIS-C features associated with severe disease requiring intensive care or treatment with biologic agents

**Fig. 2:**
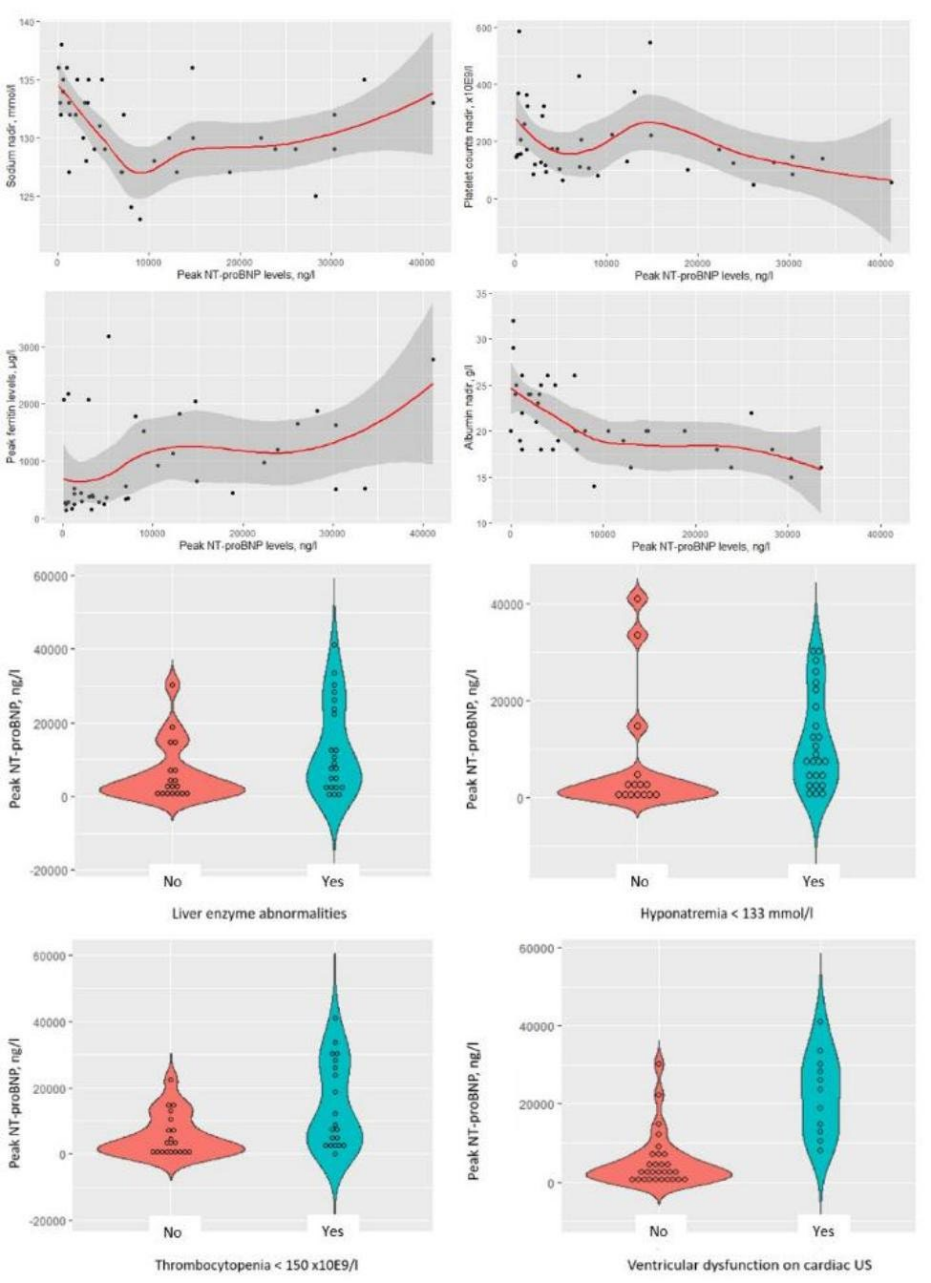
**N-terminal B-type natriuretic peptide (NT-proBNP) correlated significantly with several other MIS-C features reflecting severe disease**. **Upper panel** shows scatter plots combined with logistic regression depicting the correlation between NT-proBNP levels and sodium nadir levels, platelet counts nadir, peak ferritin levels, and albumin nadir levels, respectively. **Lower panel** depicts violin plots quantifying NT-proBNP levels in MIS-C patients with and without liver enzyme abnormalities, hyponatremia, thrombocytopenia, and ventricular dysfunction on echocardiogram, respectively

Univariate analyses revealed that intensive care need was significantly associated with the presence of circulatory shock/hypotension, laboratory evidence of (prior) SARS-CoV-2 infection, higher CRP, ferritin, D-dimer, NT-proBNP and troponin levels, lower albumin levels, ventricular dysfunction, and chest X-ray abnormalities (Table 3). Multivariate logistic regression models assessed the associations of several variables with the need for pediatric intensive care. A model encompassing NT-proBNP, CRP, and ferritin as covariates explained 70% (Nagelkerke R [2]) of the variance and correctly classified 88% of the cases. The model exploring peak NT-proBNP levels, peak ferritin levels, and circulatory shock/hypotension explained 72% of the variance in intensive care and correctly classified 88% of the cases. Finally, the model exploring peak NT-proBNP levels, peak ferritin levels, and chest X-ray abnormalities explained 89% of the variance in intensive care and correctly classified 94% of the cases. Peak NT-proBNP was the only variable significantly associated with intensive care in all multivariate models. Several logistic regression models using different combinations of the variables depicted in Table 3 were less associated with the need for intensive care compared to the models described above (data not shown). Because peak NT-proBNP levels may occur after PICU admission, we also evaluated peak levels occurring before or at time of PICU admission. Patients warranting intensive care had significantly higher NT-proBNP levels at PICU admission compared to patients managed on the general ward (median (IQR) 2107 (582–4557) ng/l versus 6022 (3367–14,852) ng/l, *U* = 91.0, *P* = 0.014). The optimal NT-proBNP cut off for PICU admission was 2406 ng/l (area under the curve 0.74, 95% CI 0.56–0.91, sensitivity 87%, specificity 52%). We explored the association of peak NT-proBNP levels with other MIS-C features (Table 4; Fig. 2). Higher peak NT-proBNP levels were associated with the presence of clinical features of shock and/or hypotension, liver enzyme abnormalities, hyponatremia, thrombocytopenia, ventricular dysfunction, aberrant findings on chest X-ray, and non-invasive and/or mechanical ventilation. Moreover, there was a poor correlation between NT-proBNP and peak CRP, peak ferritin, peak D-dimer, sodium nadir and platelets count nadir, while a moderate to good correlation was found between NT-proBNP and peak troponin and albumin nadir, respectively. Treatment with biologic agents was significantly associated with peak CRP and ferritin levels (Table 3).

**Table 3:** Distinct features of MIS-C patients warranting PICU admission or treatment with biologic agents

	PICU admission			Treatment with biologicals		
	No (n = 27)	Yes (n = 30)	*P*	No (n = 49)	Yes (n = 8)	*P*
Shock/hypotension (n, %)	20 (74)	30 (100)	**0.003**	42 (86)	8 (100)	0.58
Laboratory SARS-CoV-2 linkage (n, %)	18 (67)	29 (97)	**0.003**	41 (84)	6 (75)	0.62
Peak CRP, mg/l (median, IQR)	129.0 (57.6-234.3)	192.0 (146.6-255.9)	**0.02**	155.8 (101.0-200.7)	240.9 (132.9-319.4)	**0.02**
Peak ferritin, µg/l (median, IQR)	376 (281–434)	1356 (616–1795)	**0.001**	473 (280–926)	1380 (509–1753)	**0.05**
Peak D-dimer, mg/l (median, IQR)	2.40 (1.28–2.60)	4.81 (3.56–5.28)	**0.006**	2.44 (1.57–4.72)	4.95 (1.52–5.48)	0.32
Peak NT-proBNP, ng/l (median, IQR)	2791 (582–7010)	13,855 (8753–26,602)	**< 0.001**	4289 (1228–12,384)	19,131 (4199–29,782)	0.06
Peak troponin, ng/l (median, IQR)	17 (10–38)	42 (30–72)	**0.002**	30 (14–66)	67 (40–291)	0.07
Albumin nadir, g/l (median, IQR)	23 (20–25)	19 (16–20)	**0.03**	21 (18–26)	19 (17–22)	0.08
Ventricular dysfunction (n, %)	2 (7)	18 (60)	**< 0.001**	16 (33)	4 (50)	0.43
Chest X-ray abnormalities (n, %)	3 (11)	18 (60)	**0.002**	16 (33)	5 (63)	0.44

Variables not listed in the table lacked statistical significance. (PICU = pediatric intensive care unit, n = number, CRP = C-reactive protein, IQR = interquartile range, NT-proBNP = N-terminal B-type natriuretic peptide)

**Table 4:** Association of higher peak NT-proBNP levels with MIS-C clinical features reflecting severe disease and correlation with other MIS-C laboratory indices

	Peak NT-proBNP levels, ng/l	
	Median (IQR)	*P*
Shock/hypotension Yes No	7010 (2922–18,844) 582 (327–1095)	**0.001** (*U* = 14.0)
Liver enzyme abnormalities Yes No	8512 (2889–24,391) 3271 (867–8939)	**0.05** (*U* = 124.0)
Hyponatremia < 133 mmol/l Yes No	8031 (3570–20,578) 2107 (550–4810)	**0.02** (*U* = 101.0)
Thrombocytopenia < 150 × 10E9/l Yes No	8512 (3252–27,719) 3020 (680–9739)	**0.01** (*U* = 106.0)
Ventricular dysfunction on echocardiogram Yes No	23,840 (12,983–30,320) 3118 (1095–6981)	**< 0·001** (*U* = 20.0)
Chest X-ray abnormalities Yes No	12,217 (4957–28,182) 3115 (875-11185)	**0.02** (*U* = 72.0)
Non-invasive/mechanical ventilation Yes No	29,299 (13,093–38,446) 4290 (1228–12,792)	**0.006** (*U* = 15.0)
	**Spearman’s Rho**	***P***
Peak C-reactive protein, mg/l	0.39	**0.01**
Peak ferritin, µg/l	0.48	**0.002**
Peak D-dimer, mg/l	0.40	**0.02**
Peak troponin, ng/l	0.53	**0.001**
Sodium nadir, mmol/l	-0.43	**0.006**
Albumin nadir, g/l	-0.62	**< 0.001**
Platelet counts nadir, x10E9/l	-0.36	**0.02**

Mann-Whitney *U* values are indicated between brackets. Variables not listed in the table lacked statistical significance. (NT-proBNP = N-terminal B-type natriuretic peptide, IQR = interquartile range)

## Discussion

In the current study, we identified divergent MIS-C phenotypes across the successive waves of the COVID-19 pandemic. Collectively, MIS-C patients admitted during a later phase of the pandemic presented with distinct laboratory features, including higher ferritin, D-dimer, and NT-proBNP levels, and a higher prevalence of liver enzyme abnormalities, hypoalbuminemia, and thrombocytopenia. Moreover, there was a higher need for treatment with corticosteroids and non-invasive and/or mechanical ventilation in these patients. Of note, the threshold for starting corticosteroids was presumably lower in a later stage of the pandemic as experience with MIS-C management increased. The above findings were largely corroborated by unsupervised clustering analyses applying a self-organizing map classifying the MIS-C patients in different clusters with similar features without information regarding the wave the patients presented in for model training and optimization. In our cohort, identified risk factors for pediatric intensive care were clinical signs of circulatory shock and/or hypotension, laboratory evidence of SARS-CoV-2 exposure, hyperinflammation, high D-dimer levels, hypoalbuminemia, evidence of myocarditis, including high NT-proBNP or troponin levels and ventricular dysfunction on echocardiogram, and the presence of chest X-ray abnormalities. Of note, high NT-proBNP levels emerged as the single most pivotal factor associated with intensive care need in multivariate analyses. A NT-proBNP level of 2406 ng/l was identified as the cut off with the highest sensitivity and specificity.

Interestingly, distinct MIS-C phenotypes were noted across the different phases of the pandemic, presumably representing the impact of particular COVID-19 variants. MIS-C patients presenting during the first two waves of the pandemic were characterized by a relatively high prevalence of KD-like mucocutaneous features. Strikingly, the alpha and delta variant, main drivers of the third and fourth wave in Canada, seemed to trigger a more severe MIS-C phenotype including a higher frequency of macrophage activation syndrome (MAS)-like features, such as hyperferritinemia, coagulopathy, aberrant liver enzymes, and thrombocytopenia [16, 17]. These laboratory findings are acknowledged to be associated with MIS-C [6, 7, 17], but the observed shift towards a preeminence of these features during the third and especially the fourth wave of the pandemic has never been described before. Rodriguez-Smith et al. compared the levels of several serum inflammatory biomarkers of interest in patients with MIS-C, KD, and MAS, demonstrating that MIS-C, unlike KD, was characterized by higher serum levels of CXCL9, an important cytokine in the pathophysiology of MAS [17]. CXCL9 levels matched those of MAS patients, obscuring the distinction between both disease entities. Interestingly, Rodriguez-Smith et al. stratified MIS-C patients based on CXCL9 serum levels [18]. Patients with high CXCL9 levels suffered from more severe disease including a higher prevalence of circulatory shock, myocardial dysfunction, coagulopathy, cytopenia, and higher inflammatory markers compared to patients with low serum levels. The latter group had a phenotype resembling KD patients. Interferon ƴ-induced CXCL may therefore have played a more important pathophysiological role in later stages of the pandemic. Future research assessing inflammatory cytokine profiles in MIS-C patients across the successive COVID-19 waves may reflect the observed shift in phenotype.

High serum NT-proBNP levels heavily impact MIS-C prognosis, as they are associated with a greater disease burden, reflected by the presence of hypotension and/or circulatory shock, hyperinflammation, hypoalbuminemia, ventricular dysfunction on echocardiogram, chest X-ray abnormalities, and the need for pediatric intensive care. Concordantly, in a recent study by Abrams et al. in 1080 MIS-C patients, the presence of increased NT-proBNP levels was strongly associated with intensive care unit admission [10]. Other identified risk factors were age 6–12 years and especially 13–20 years, non-Hispanic Black ethnicity, shortness of breath, abdominal pain, thrombocytopenia, lymphocytopenia, and increased levels of CRP, troponin, ferritin, D-dimers or IL-6. Our study results largely corroborate the findings of Abrams et al. Moreover, the present study underscores the important role of NT-proBNP in these and its association with other MIS-C features reflecting more severe disease. Intriguingly, the risk factors for severe MIS-C are in stark contrast with those for children with acute COVID-19, as these include hypoxia on admission, viral coinfections, underlying chronic comorbidities, obesity, lymphocytopenia, and moderate to severe liver disease [19–21]. While MIS-C mainly affects previously healthy children [7, 22], severe acute SARS-CoV-2 infection is often seen in children with pre-existing clinical conditions, such as preterm birth, asthma, and immune deficiency [23]. These findings point towards the involvement of different pathophysiologic mechanisms in both disease entities. Of note, our results underscore the importance of monitoring serum NT-proBNP levels in MIS-C patients. Rising values warrant repeat echocardiogram and close monitoring.

There are several limitations to the present study. The number of MIS-C patients in the first and fourth wave of the COVID-19 pandemic were small, limiting the detection of relevant differences between the respective waves. Nevertheless, despite these small numbers, several significant and important differences were observed. The classification of MIS-C patients in different waves may be arbitrary, as the waves and different SARS-CoV-2 variants may have overlapped. The clustering analysis confirmed this limitation as miss-classified cases often presented in the previous or subsequent wave. Considering the relative small absolute number of patients needing intensive care, multivariate analyses were restricted to the use of three covariates. Finally, we have no data about the prevalence of MIS-C in smaller regional hospitals in Alberta, Canada. It may be plausible that in later stages of the pandemic patients with less severe disease were less often transferred to tertiary hospitals compared to the first waves. Strengths of our study include the homogeneity in assessing, monitoring and managing patients, and capturing the data, as the study was restricted to a single tertiary center.

## Conclusion

Collectively, MIS-C patients presenting in a later stage of the COVID-19 pandemic displayed a distinct and more severe phenotype characterized by a predominance of MAS-like features, including higher ferritin and D-dimer levels, liver enzyme abnormalities, and thrombocytopenia. This finding presumably reflects the impact of distinct SARS-CoV-2 variants. High NT-proBNP levels emerged as an important feature associated with the need for intensive care. Future studies could evaluate the pathophysiologic differences in these distinct phenotypes by assessing inflammatory cytokine profiles in MIS-C patients across the COVID-19 waves and in patients with high versus low NT-proBNP levels.

## Electronic supplementary material

Below is the link to the electronic supplementary material.

Supplementary Material 1

## Data Availability

The datasets used and/or analyzed during the current study are available from the corresponding author on reasonable request.

## Abbreviations

SARS-CoV-2: severe acute respiratory syndrome coronavirus 2
COVID-19: coronavirus disease 2019
MIS-C: Multisystem inflammatory syndrome in children
KD: Kawasaki disease
PICU: pediatric intensive care unit
CRP: C-reactive protein
NT-proBNP: N-terminal B-type natriuretic peptide
IL: interleukin
IVIG: intravenous immunoglobulins
AHA: American Heart Association
IQR: interquartile range
TNF: tumor necrosis factor
n: number
INR: international normalized ratio
US: ultrasound
CPAP: continuous positive airway pressure
BiPAP: bilevel positive airway pressure
MAS: macrophage activation syndrome
