# Supplementary material for: Distinct phenotypes of multisystem inflammatory syndrome in children: a cohort study
Source: Pediatr Rheumatol Online J. 2023 Apr 12;21:33. doi: 10.1186/s12969-023-00815-w (PMC10092941; doi:10.1186/s12969-023-00815-w)
Supplement: Supplementary file 1 — Supplementary Material 1 [file 12969_2023_815_MOESM1_ESM.docx]

eTable 1: The unsupervised clustering model applying a self-organizing map grouped MIS-C patients in four (upper panel) and two (lower panel) clusters based on similar features.

|  | **Cluster 1**  (n = 8) | **Cluster 2**  (n = 7) | **Cluster 3**  (n = 22) | **Cluster 4**  (n = 20) |
| --- | --- | --- | --- | --- |
| Patients, n (%):  Wave 1  Wave 2  Wave 3  Wave 4 | 1 (13)  0  5 (63)  2 (25) | 0  3 (43)  2 (29)  2 (29) | 0  14 (64)  7 (32)  1 (5) | 6 (30)  8 (40)  3 (15)  3 (15) |
| Age, years | 8 | 4 | 6 | 6 |
| Fulfillment of AHA KD criteria | 0.38 | 0.29 | 0.50 | 0.30 |
| Days of fever at admission | 4 | 4 | 5 | 6 |
| Extremity changes | 0.88 | 0.57 | 0.77 | 0.65 |
| Skin rash | 0.88 | 0.71 | 0.95 | 0.75 |
| Conjunctivitis | 1 | 1 | 0.95 | 0.85 |
| Changes lips and/or oral cavity | 0.75 | 0.71 | 0.77 | 0.75 |
| Cervical lymphadenopathy | 0.13 | 0.14 | 0.23 | 0.10 |
| Gastrointestinal manifestations | 0.88 | 1 | 0.95 | 1 |
| Shock/hypotension | 1 | 1 | 0.91 | 0.75 |
| Coagulation dysfunction | 0.88 | 1 | 0.77 | 0.80 |
| Peak C-reactive protein, mg/l | 235.0 | 174.4 | 157.0 | 141.1 |
| Peak ferritin, μg/l | 1390 | 1234 | 695 | 575 |
| Peak D-dimer, mg/l | 4.18 | 2.78 | 3.70 | 2.53 |
| Peak troponin, ng/l | 77.1 | 198.2 | 120.9 | 322.0 |
| Peak NT-proBNP, ng/l | 29475 | 7696 | 14724 | 1979 |
| Liver enzyme abnormalities | 0.88 | 0.71 | 0.36 | 0.40 |
| Hyponatremia | 0.75 | 1 | 0.68 | 0.40 |
| Sodium nadir, mmol/l | 130 | 127 | 131 | 133 |
| Hypoalbuminemia | 1 | 1 | 0.77 | 0.95 |
| Albumin nadir, g/l | 17 | 20 | 23 | 23 |
| Thrombocytopenia | 0.88 | 0.57 | 0.50 | 0.35 |
| Platelet count nadir, x10E9/l | 113 | 175 | 198 | 218 |
| Concurrent other viral infection | 0 | 0.29 | 0 | 0.10 |
| Concurrent bacterial infection | 0 | 0.14 | 0.05 | 0 |
| Coronary abnormalities | 0.25 | 0 | 0.09 | 0.20 |
| Ventricular dysfunction | 0.75 | 0.29 | 0.55 | 0 |
| Chest X-ray abnormalities | 0.63 | 0.50 | 0.67 | 0.18 |
|  | | | | |
|  | **Cluster 1**  (n = 28) | | **Cluster 2**  (n = 29) | |
| Patients, n (%):  Phase 1  Phase 2 | 9 (32)  19 (68) | | 23 (79)  6 (21) | |
| Age, years | 7 | | 5 | |
| Fulfillment of AHA KD criteria | 0.43 | | 0.34 | |
| Days of fever at admission | 5 | | 5 | |
| Extremity changes | 0.71 | | 0.72 | |
| Skin rash | 0.79 | | 0.90 | |
| Conjunctivitis | 0.93 | | 0.93 | |
| Changes lips and/or oral cavity | 0.68 | | 0.76 | |
| Cervical lymphadenopathy | 0.21 | | 0.10 | |
| Gastrointestinal manifestations | 0.93 | | 1 | |
| Shock/hypotension | 0.93 | | 0.83 | |
| Coagulation dysfunction | 0.86 | | 0.79 | |
| Peak C-reactive protein, mg/l | 198.3 | | 131.9 | |
| Peak ferritin, μg/l | 1311 | | 340 | |
| Peak D-dimer, mg/l | 4.69 | | 2.04 | |
| Peak troponin, ng/l | 123.9 | | 273.4 | |
| Peak NT-proBNP, ng/l | 16498 | | 3646 | |
| Liver enzyme abnormalities | 0.64 | | 0.34 | |
| Hyponatremia | 0.68 | | 0.59 | |
| Sodium nadir, mmol/l | 130 | | 132 | |
| Hypoalbuminemia | 1 | | 0.79 | |
| Albumin nadir, g/l | 19 | | 25 | |
| Thrombocytopenia | 0.64 | | 0.38 | |
| Platelet count nadir, x10E9/l | 155 | | 224 | |
| Concurrent other viral infection | 0.04 | | 0.10 | |
| Concurrent bacterial infection | 0.04 | | 0.04 | |
| Coronary abnormalities | 0.07 | | 0.21 | |
| Ventricular dysfunction | 0.50 | | 0.21 | |
| Chest X-ray abnormalities | 0.60 | | 0.29 | |

Values in the table are displayed as mean.
